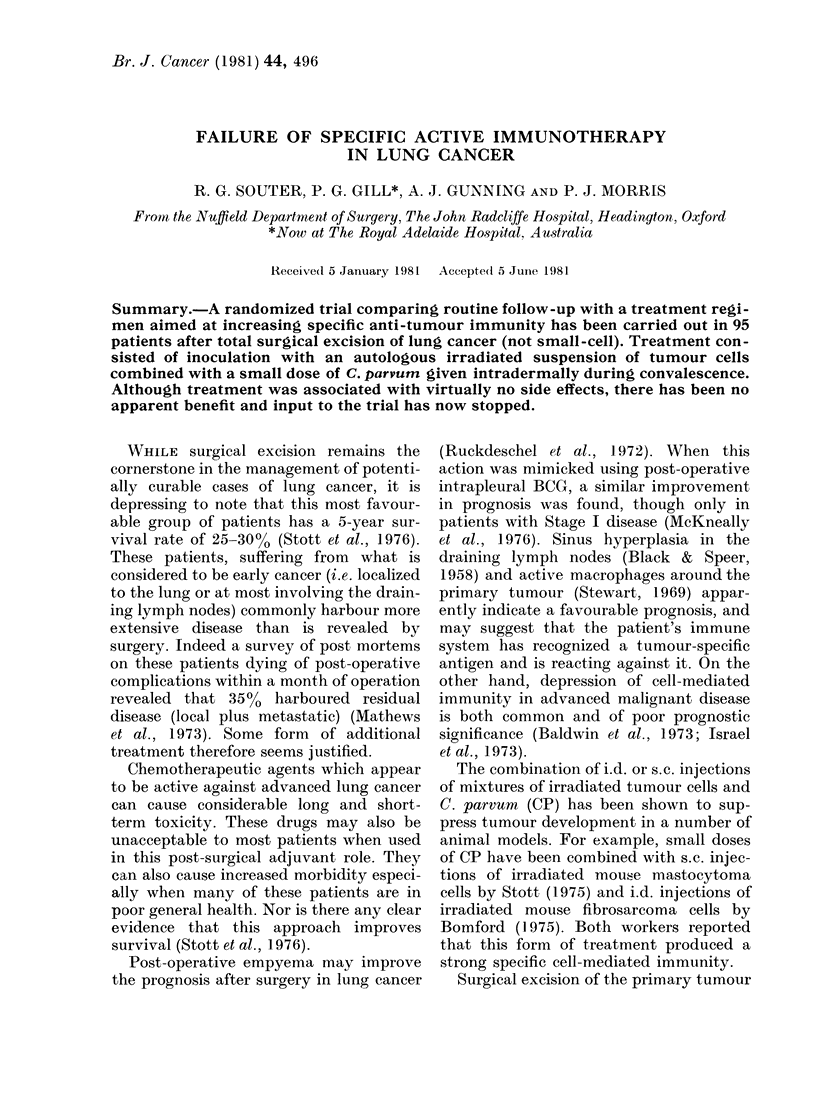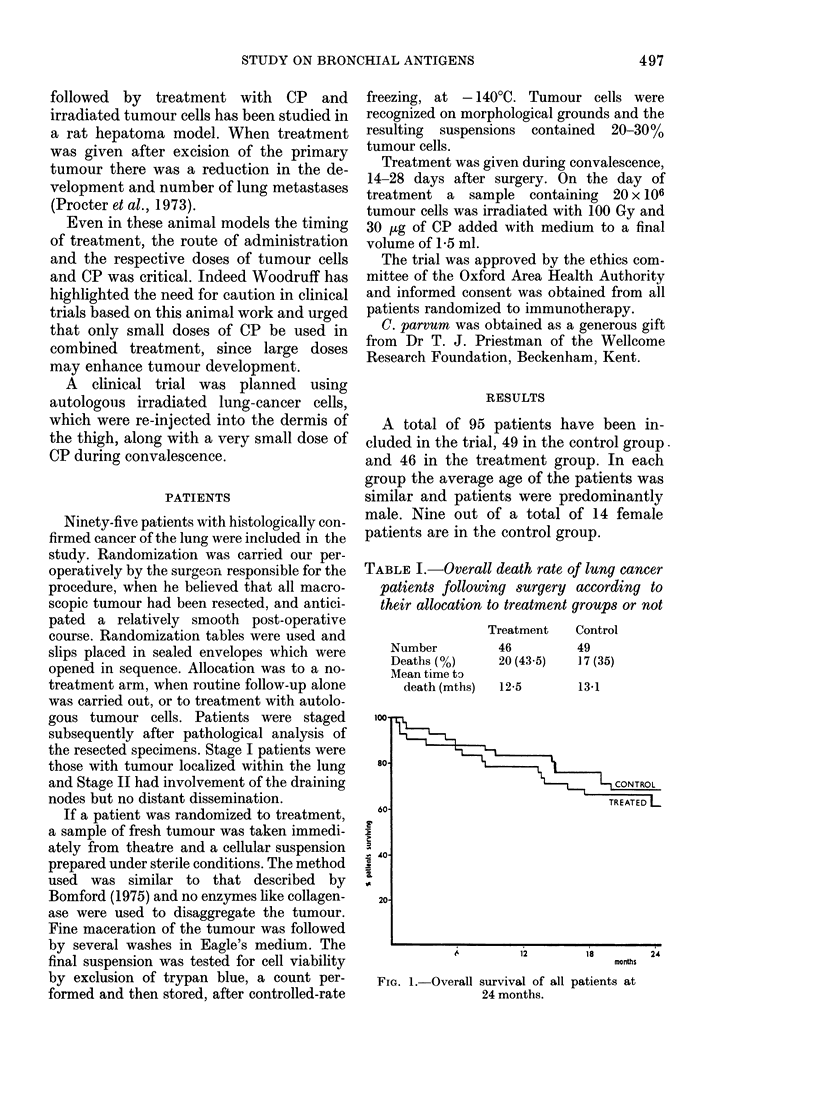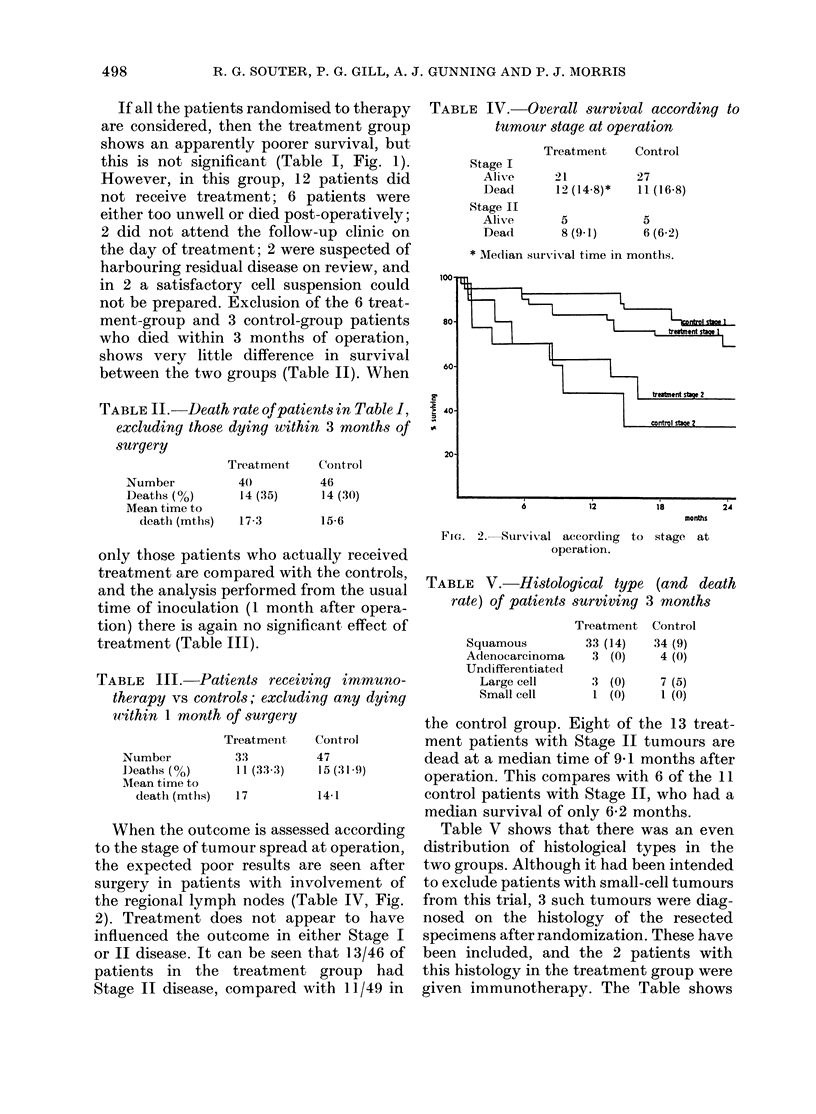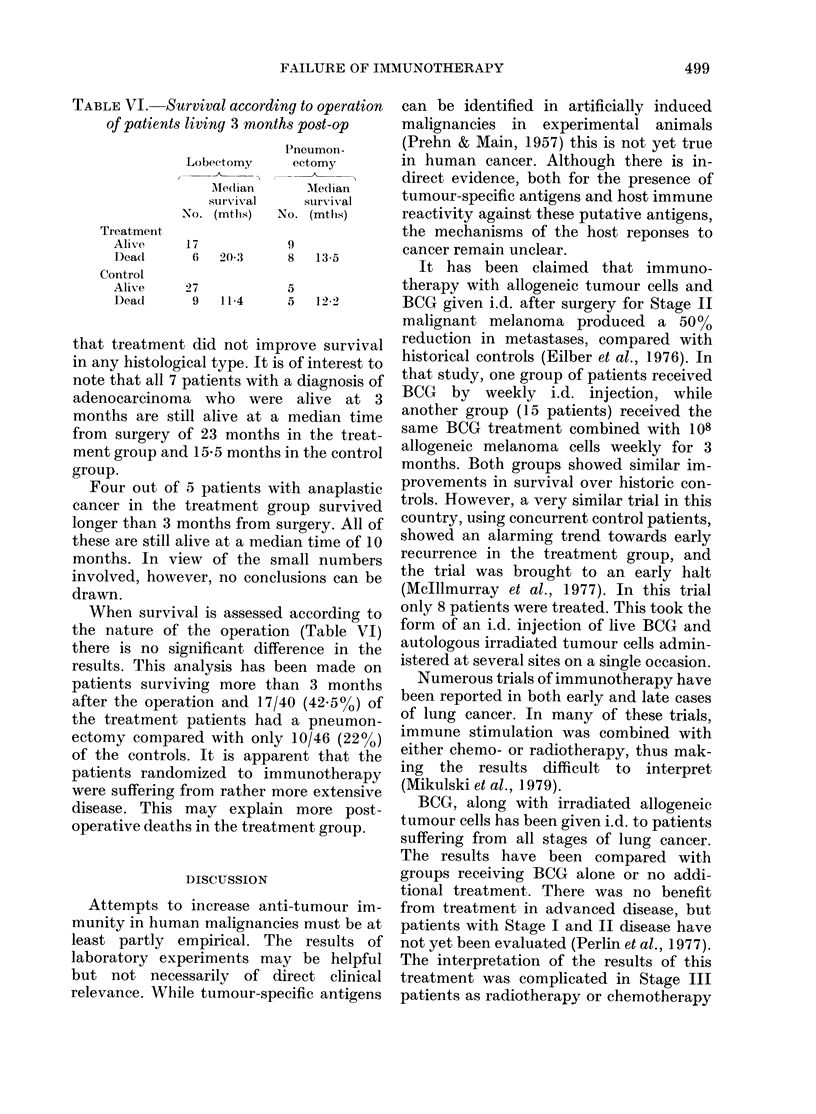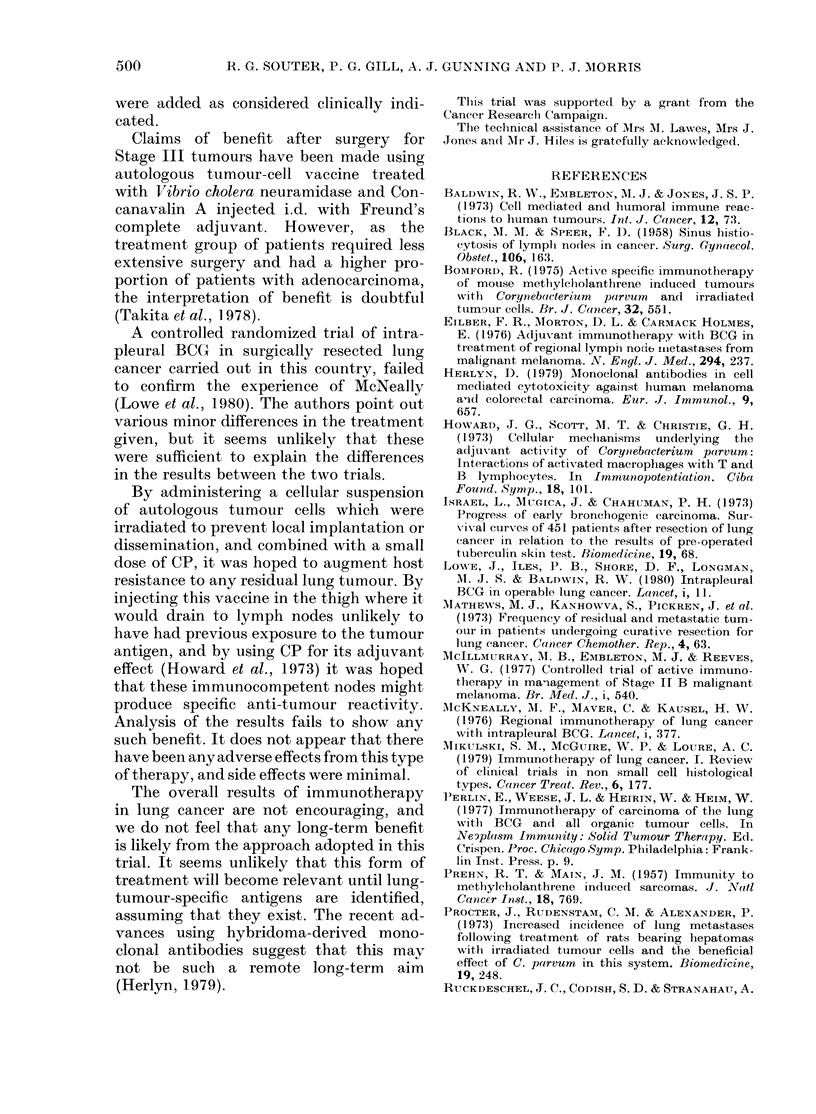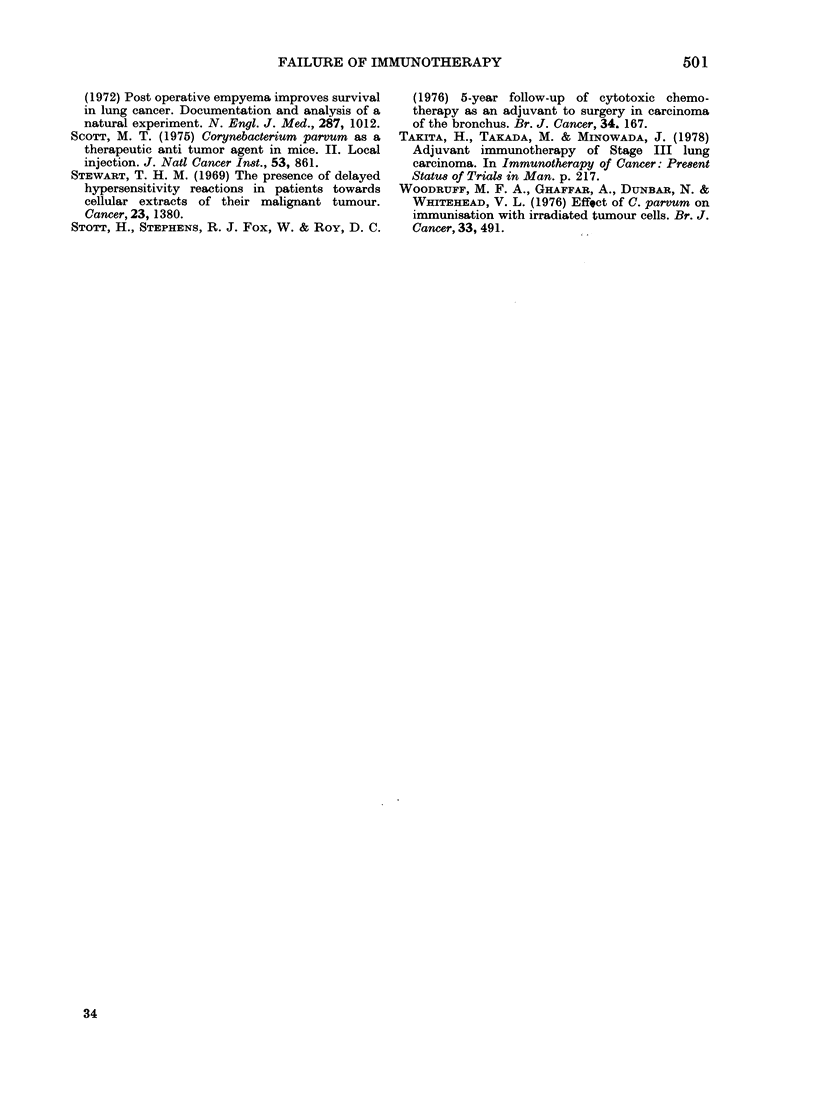# Failure of specific active immunotherapy in lung cancer.

**DOI:** 10.1038/bjc.1981.217

**Published:** 1981-10

**Authors:** R. G. Souter, P. G. Gill, A. J. Gunning, P. J. Morris

## Abstract

A randomized trial comparing routine follow-up with a treatment regimen aimed at increasing specific anti-tumour immunity has been carried out in 95 patients after total surgical excision of lung cancer (not small-cell). Treatment consisted of inoculation with an autologous irradiated suspension of tumour cells combined with a small dose of C. parvum given intradermally during convalescence. Although treatment was associated with virtually no side effects, there has been no apparent benefit and input to the trial has now stopped.


*Br. J. Cancer* (1981) **44**, 496

### FAILURE OF SPECIFIC ACTIVE IMMUNOTHERAPY IN LUNG CANCER

R. G. SOUTER, P. G. GILL*, A. J. GUNNING AND P. J. MORRIS

*From the Nuffield Department of Surgery, The John Radcliffe Hospital, Headington, Oxford*
**Now at The Royal Adelaide Hospital, Australia*

Received 5 January 1981 Accepted 5 June 1981

**Summary.—A randomized trial comparing routine follow-up with a treatment regimen aimed at increasing specific anti-tumour immunity has been carried out in 95 patients after total surgical excision of lung cancer (not small-cell). Treatment consisted of inoculation with an autologous irradiated suspension of tumour cells combined with a small dose of *C. parvum* given intradermally during convalescence. Although treatment was associated with virtually no side effects, there has been no apparent benefit and input to the trial has now stopped.**

WHILE surgical excision remains the cornerstone in the management of potentially curable cases of lung cancer, it is depressing to note that this most favourable group of patients has a 5-year survival rate of 25–30% (Stott *et al.*, 1976). These patients, suffering from what is considered to be early cancer (*i.e.* localized to the lung or at most involving the draining lymph nodes) commonly harbour more extensive disease than is revealed by surgery. Indeed a survey of post mortems on these patients dying of post-operative complications within a month of operation revealed that 35% harboured residual disease (local plus metastatic) (Mathews *et al.*, 1973). Some form of additional treatment therefore seems justified.

Chemotherapeutic agents which appear to be active against advanced lung cancer can cause considerable long and short-term toxicity. These drugs may also be unacceptable to most patients when used in this post-surgical adjuvant role. They can also cause increased morbidity especially when many of these patients are in poor general health. Nor is there any clear evidence that this approach improves survival (Stott *et al.*, 1976).

Post-operative empyema may improve the prognosis after surgery in lung cancer (Ruckdeschel *et al.*, 1972). When this action was mimicked using post-operative intrapleural BCG, a similar improvement in prognosis was found, though only in patients with Stage I disease (McKneally *et al.*, 1976). Sinus hyperplasia in the draining lymph nodes (Black & Speer, 1958) and active macrophages around the primary tumour (Stewart, 1969) apparently indicate a favourable prognosis, and may suggest that the patient's immune system has recognized a tumour-specific antigen and is reacting against it. On the other hand, depression of cell-mediated immunity in advanced malignant disease is both common and of poor prognostic significance (Baldwin *et al.*, 1973; Israel *et al.*, 1973).

The combination of i.d. or s.c. injections of mixtures of irradiated tumour cells and *C. parvum* (CP) has been shown to suppress tumour development in a number of animal models. For example, small doses of CP have been combined with s.c. injections of irradiated mouse mastocytoma cells by Stott (1975) and i.d. injections of irradiated mouse fibrosarcoma cells by Bomford (1975). Both workers reported that this form of treatment produced a strong specific cell-mediated immunity.

Surgical excision of the primary tumour followed by treatment with CP and irradiated tumour cells has been studied in a rat hepatoma model. When treatment was given after excision of the primary tumour there was a reduction in the development and number of lung metastases (Procter *et al.*, 1973).

Even in these animal models the timing of treatment, the route of administration and the respective doses of tumour cells and CP was critical. Indeed Woodruff has highlighted the need for caution in clinical trials based on this animal work and urged that only small doses of CP be used in combined treatment, since large doses may enhance tumour development.

A clinical trial was planned using autologous irradiated lung-cancer cells, which were re-injected into the dermis of the thigh, along with a very small dose of CP during convalescence.

### PATIENTS

Ninety-five patients with histologically confirmed cancer of the lung were included in the study. Randomization was carried our peroperatively by the surgeon responsible for the procedure, when he believed that all macroscopic tumour had been resected, and anticipated a relatively smooth post-operative course. Randomization tables were used and slips placed in sealed envelopes which were opened in sequence. Allocation was to a no-treatment arm, when routine follow-up alone was carried out, or to treatment with autologous tumour cells. Patients were staged subsequently after pathological analysis of the resected specimens. Stage I patients were those with tumour localized within the lung and Stage II had involvement of the draining nodes but no distant dissemination.

If a patient was randomized to treatment, a sample of fresh tumour was taken immediately from theatre and a cellular suspension prepared under sterile conditions. The method used was similar to that described by Bomford (1975) and no enzymes like collagenase were used to disaggregate the tumour. Fine maceration of the tumour was followed by several washes in Eagle's medium. The final suspension was tested for cell viability by exclusion of trypan blue, a count performed and then stored, after controlled-rate freezing, at $-140°C$. Tumour cells were recognized on morphological grounds and the resulting suspensions contained 20–30% tumour cells.

Treatment was given during convalescence, 14–28 days after surgery. On the day of treatment a sample containing $20 \times 10^6$ tumour cells was irradiated with 100 Gy and 30 $\mu g$ of CP added with medium to a final volume of 1·5 ml.

The trial was approved by the ethics committee of the Oxford Area Health Authority and informed consent was obtained from all patients randomized to immunotherapy.

*C. parvum* was obtained as a generous gift from Dr T. J. Priestman of the Wellcome Research Foundation, Beckenham, Kent.

### RESULTS

A total of 95 patients have been included in the trial, 49 in the control group and 46 in the treatment group. In each group the average age of the patients was similar and patients were predominantly male. Nine out of a total of 14 female patients are in the control group.

TABLE I.—*Overall death rate of lung cancer patients following surgery according to their allocation to treatment groups or not*

| | Treatment | Control |
|---|---|---|
| Number | 46 | 49 |
| Deaths (%) | 20 (43·5) | 17 (35) |
| Mean time to death (mths) | 12·5 | 13·1 |

FIG. 1.—Overall survival of all patients at 24 months.

If all the patients randomised to therapy are considered, then the treatment group shows an apparently poorer survival, but this is not significant (Table I, Fig. 1). However, in this group, 12 patients did not receive treatment; 6 patients were either too unwell or died post-operatively; 2 did not attend the follow-up clinic on the day of treatment; 2 were suspected of harbouring residual disease on review, and in 2 a satisfactory cell suspension could not be prepared. Exclusion of the 6 treatment-group and 3 control-group patients who died within 3 months of operation, shows very little difference in survival between the two groups (Table II). When only those patients who actually received treatment are compared with the controls, and the analysis performed from the usual time of inoculation (1 month after operation) there is again no significant effect of treatment (Table III).

TABLE II.—*Death rate of patients in Table I, excluding those dying within 3 months of surgery*

| | Treatment | Control |
|---|---|---|
| Number | 40 | 46 |
| Deaths (%) | 14 (35) | 14 (30) |
| Mean time to death (mths) | 17·3 | 15·6 |

TABLE III.—*Patients receiving immunotherapy* vs *controls; excluding any dying within* 1 *month of surgery*

| | Treatment | Control |
|---|---|---|
| Number | 33 | 47 |
| Deaths (%) | 11 (33·3) | 15 (31·9) |
| Mean time to death (mths) | 17 | 14·1 |

When the outcome is assessed according to the stage of tumour spread at operation, the expected poor results are seen after surgery in patients with involvement of the regional lymph nodes (Table IV, Fig. 2). Treatment does not appear to have influenced the outcome in either Stage I or II disease. It can be seen that 13/46 of patients in the treatment group had Stage II disease, compared with 11/49 in the control group. Eight of the 13 treatment patients with Stage II tumours are dead at a median time of 9·1 months after operation. This compares with 6 of the 11 control patients with Stage II, who had a median survival of only 6·2 months.

TABLE IV.—*Overall survival according to tumour stage at operation*

| | Treatment | Control |
|---|---|---|
| Stage I | | |
| Alive | 21 | 27 |
| Dead | 12 (14·8)* | 11 (16·8) |
| Stage II | | |
| Alive | 5 | 5 |
| Dead | 8 (9·1) | 6 (6·2) |

* Median survival time in months.

FIG. 2.—Survival according to stage at operation.

TABLE V.—*Histological type (and death rate) of patients surviving* 3 *months*

| | Treatment | Control |
|---|---|---|
| Squamous | 33 (14) | 34 (9) |
| Adenocarcinoma | 3 (0) | 4 (0) |
| Undifferentiated | | |
| Large cell | 3 (0) | 7 (5) |
| Small cell | 1 (0) | 1 (0) |

Table V shows that there was an even distribution of histological types in the two groups. Although it had been intended to exclude patients with small-cell tumours from this trial, 3 such tumours were diagnosed on the histology of the resected specimens after randomization. These have been included, and the 2 patients with this histology in the treatment group were given immunotherapy. The Table shows

TABLE VI.—*Survival according to operation of patients living 3 months post-op*

| | Lobectomy | | Pneumonectomy | |
|---|---|---|---|---|
| | No. | Median survival (mths) | No. | Median survival (mths) |
| Treatment | | | | |
| Alive | 17 | | 9 | |
| Dead | 6 | 20·3 | 8 | 13·5 |
| Control | | | | |
| Alive | 27 | | 5 | |
| Dead | 9 | 11·4 | 5 | 12·2 |

that treatment did not improve survival in any histological type. It is of interest to note that all 7 patients with a diagnosis of adenocarcinoma who were alive at 3 months are still alive at a median time from surgery of 23 months in the treatment group and 15·5 months in the control group.

Four out of 5 patients with anaplastic cancer in the treatment group survived longer than 3 months from surgery. All of these are still alive at a median time of 10 months. In view of the small numbers involved, however, no conclusions can be drawn.

When survival is assessed according to the nature of the operation (Table VI) there is no significant difference in the results. This analysis has been made on patients surviving more than 3 months after the operation and 17/40 (42·5%) of the treatment patients had a pneumonectomy compared with only 10/46 (22%) of the controls. It is apparent that the patients randomized to immunotherapy were suffering from rather more extensive disease. This may explain more post-operative deaths in the treatment group.

### DISCUSSION

Attempts to increase anti-tumour immunity in human malignancies must be at least partly empirical. The results of laboratory experiments may be helpful but not necessarily of direct clinical relevance. While tumour-specific antigens can be identified in artificially induced malignancies in experimental animals (Prehn & Main, 1957) this is not yet true in human cancer. Although there is indirect evidence, both for the presence of tumour-specific antigens and host immune reactivity against these putative antigens, the mechanisms of the host reponses to cancer remain unclear.

It has been claimed that immunotherapy with allogeneic tumour cells and BCG given i.d. after surgery for Stage II malignant melanoma produced a 50% reduction in metastases, compared with historical controls (Eilber *et al.*, 1976). In that study, one group of patients received BCG by weekly i.d. injection, while another group (15 patients) received the same BCG treatment combined with $10^8$ allogeneic melanoma cells weekly for 3 months. Both groups showed similar improvements in survival over historic controls. However, a very similar trial in this country, using concurrent control patients, showed an alarming trend towards early recurrence in the treatment group, and the trial was brought to an early halt (McIllmurray *et al.*, 1977). In this trial only 8 patients were treated. This took the form of an i.d. injection of live BCG and autologous irradiated tumour cells administered at several sites on a single occasion.

Numerous trials of immunotherapy have been reported in both early and late cases of lung cancer. In many of these trials, immune stimulation was combined with either chemo- or radiotherapy, thus making the results difficult to interpret (Mikulski *et al.*, 1979).

BCG, along with irradiated allogeneic tumour cells has been given i.d. to patients suffering from all stages of lung cancer. The results have been compared with groups receiving BCG alone or no additional treatment. There was no benefit from treatment in advanced disease, but patients with Stage I and II disease have not yet been evaluated (Perlin *et al.*, 1977). The interpretation of the results of this treatment was complicated in Stage III patients as radiotherapy or chemotherapy were added as considered clinically indicated.

Claims of benefit after surgery for Stage III tumours have been made using autologous tumour-cell vaccine treated with *Vibrio cholera* neuramidase and Concanavalin A injected i.d. with Freund's complete adjuvant. However, as the treatment group of patients required less extensive surgery and had a higher proportion of patients with adenocarcinoma, the interpretation of benefit is doubtful (Takita *et al.*, 1978).

A controlled randomized trial of intrapleural BCG in surgically resected lung cancer carried out in this country, failed to confirm the experience of McNeally (Lowe *et al.*, 1980). The authors point out various minor differences in the treatment given, but it seems unlikely that these were sufficient to explain the differences in the results between the two trials.

By administering a cellular suspension of autologous tumour cells which were irradiated to prevent local implantation or dissemination, and combined with a small dose of CP, it was hoped to augment host resistance to any residual lung tumour. By injecting this vaccine in the thigh where it would drain to lymph nodes unlikely to have had previous exposure to the tumour antigen, and by using CP for its adjuvant effect (Howard *et al.*, 1973) it was hoped that these immunocompetent nodes might produce specific anti-tumour reactivity. Analysis of the results fails to show any such benefit. It does not appear that there have been any adverse effects from this type of therapy, and side effects were minimal.

The overall results of immunotherapy in lung cancer are not encouraging, and we do not feel that any long-term benefit is likely from the approach adopted in this trial. It seems unlikely that this form of treatment will become relevant until lung-tumour-specific antigens are identified, assuming that they exist. The recent advances using hybridoma-derived monoclonal antibodies suggest that this may not be such a remote long-term aim (Herlyn, 1979).

This trial was supported by a grant from the Cancer Research Campaign.

The technical assistance of Mrs M. Lawes, Mrs J. Jones and Mr J. Hiles is gratefully acknowledged.

### REFERENCES

Baldwin, R. W., Embleton, M. J. & Jones, J. S. P. (1973) Cell mediated and humoral immune reactions to human tumours. *Int. J. Cancer*, **12**, 73.

Black, M. M. & Speer, F. D. (1958) Sinus histiocytosis of lymph nodes in cancer. *Surg. Gynaecol. Obstet.*, **106**, 163.

Bomford, R. (1975) Active specific immunotherapy of mouse methylcholanthrene induced tumours with *Corynebacterium parvum* and irradiated tumour cells. *Br. J. Cancer*, **32**, 551.

Eilber, F. R., Morton, D. L. & Carmack Holmes, E. (1976) Adjuvant immunotherapy with BCG in treatment of regional lymph node metastases from malignant melanoma. *N. Engl. J. Med.*, **294**, 237.

Herlyn, D. (1979) Monoclonal antibodies in cell mediated cytotoxicity against human melanoma and colorectal carcinoma. *Eur. J. Immunol.*, **9**, 657.

Howard, J. G., Scott, M. T. & Christie, G. H. (1973) Cellular mechanisms underlying the adjuvant activity of *Corynebacterium parvum*: Interactions of activated macrophages with T and B lymphocytes. In *Immunopotentiation. Ciba Found. Symp.*, **18**, 101.

Israel, L., Mugica, J. & Chahuman, P. H. (1973) Progress of early bronchogenic carcinoma. Survival curves of 451 patients after resection of lung cancer in relation to the results of pre-operated tuberculin skin test. *Biomedicine*, **19**, 68.

Lowe, J., Iles, P. B., Shore, D. F., Longman, M. J. S. & Baldwin, R. W. (1980) Intrapleural BCG in operable lung cancer. *Lancet*, i, 11.

Mathews, M. J., Kanhowva, S., Pickren, J. *et al.* (1973) Frequency of residual and metastatic tumour in patients undergoing curative resection for lung cancer. *Cancer Chemother. Rep.*, **4**, 63.

McIllmurray, M. B., Embleton, M. J. & Reeves, W. G. (1977) Controlled trial of active immunotherapy in management of Stage II B malignant melanoma. *Br. Med. J.*, i, 540.

McKneally, M. F., Maver, C. & Kausel, H. W. (1976) Regional immunotherapy of lung cancer with intrapleural BCG. *Lancet*, i, 377.

Mikulski, S. M., McGuire, W. P. & Loure, A. C. (1979) Immunotherapy of lung cancer. I. Review of clinical trials in non small cell histological types. *Cancer Treat. Rev.*, **6**, 177.

Perlin, E., Weese, J. L. & Heirin, W. & Heim, W. (1977) Immunotherapy of carcinoma of the lung with BCG and all organic tumour cells. In *Neoplasm Immunity: Solid Tumour Therapy*. Ed. Crispen. *Proc. Chicago Symp.* Philadelphia: Franklin Inst. Press. p. 9.

Prehn, R. T. & Main, J. M. (1957) Immunity to methylcholanthrene induced sarcomas. *J. Natl Cancer Inst.*, **18**, 769.

Procter, J., Rudenstam, C. M. & Alexander, P. (1973) Increased incidence of lung metastases following treatment of rats bearing hepatomas with irradiated tumour cells and the beneficial effect of *C. parvum* in this system. *Biomedicine*, **19**, 248.

Ruckdeschel, J. C., Codish, S. D. & Stranahau, A.

(1972) Post operative empyema improves survival in lung cancer. Documentation and analysis of a natural experiment. *N. Engl. J. Med.*, **287**, 1012.

SCOTT, M. T. (1975) *Corynebacterium parvum* as a therapeutic anti tumor agent in mice. II. Local injection. *J. Natl Cancer Inst.*, **53**, 861.

STEWART, T. H. M. (1969) The presence of delayed hypersensitivity reactions in patients towards cellular extracts of their malignant tumour. *Cancer*, **23**, 1380.

STOTT, H., STEPHENS, R. J. FOX, W. & ROY, D. C. (1976) 5-year follow-up of cytotoxic chemotherapy as an adjuvant to surgery in carcinoma of the bronchus. *Br. J. Cancer*, **34**. 167.

TAKITA, H., TAKADA, M. & MINOWADA, J. (1978) Adjuvant immunotherapy of Stage III lung carcinoma. In *Immunotherapy of Cancer: Present Status of Trials in Man*. p. 217.

WOODRUFF, M. F. A., GHAFFAR, A., DUNBAR, N. & WHITEHEAD, V. L. (1976) Effect of *C. parvum* on immunisation with irradiated tumour cells. *Br. J. Cancer*, **33**, 491.